# Plasmon Modulated Upconversion Biosensors

**DOI:** 10.3390/bios13030306

**Published:** 2023-02-22

**Authors:** Anara Molkenova, Hye Eun Choi, Jeong Min Park, Jin-Ho Lee, Ki Su Kim

**Affiliations:** 1 Institute of Advanced Organic Materials, Pusan National University, 2 Busandaehak-ro 63 beon-gil, Geumjeong-gu, Busan 46241, Republic of Korea; 2 School of Chemical Engineering, College of Engineering, Pusan National University, 2 Busandaehak-ro 63 beon-gil, Geumjeong-gu, Busan 46241, Republic of Korea; 3 School of Biomedical Convergence Engineering, Pusan National University, 49 Busandaehak-ro, Yangsan 50612, Republic of Korea; 4 Department of Organic Material Science & Engineering, Pusan National University, 2 Busandaehak-ro 63 beon-gil, Geumjeong-gu, Busan 46241, Republic of Korea

**Keywords:** upconversion nanoparticles (UCNPs), biosensing, plasmon-enhanced upconversion, plasmon modulated upconversion, upconversion quenching, plasmonic nanoparticles (PNPs), gold nanoparticles (GNPs), surface plasmon resonance (SPR), fluorescence resonance energy transfer (FRET)

## Abstract

Over the past two decades, lanthanide-based upconversion nanoparticles (UCNPs) have been fascinating scientists due to their ability to offer unprecedented prospects to upconvert tissue-penetrating near-infrared light into color-tailorable optical illumination inside biological matter. In particular, luminescent behavior UCNPs have been widely utilized for background-free biorecognition and biosensing. Currently, a paramount challenge exists on how to maximize NIR light harvesting and upconversion efficiencies for achieving faster response and better sensitivity without damaging the biological tissue upon laser assisted photoactivation. In this review, we offer the reader an overview of the recent updates about exciting achievements and challenges in the development of plasmon-modulated upconversion nanoformulations for biosensing application.

## 1. Introduction

Lanthanide-based upconversion nanoparticles (UCNPs) have become the engine of numerous ground-breaking inventions in a wide variety of research areas. By disobeying the Stokes Law, UCNPs are capable of producing higher energy output photons out of multiple (>2) lower energy input photons. For example, one can obtain ultraviolet (UV) or visible emission by exposing UCNPs to near infrared (NIR) laser. Contrary to other light upconverting analogues, such as organic luminophores and quantum dots, lanthanide-based UCNPs indeed possess overwhelming advantages, which include a diversity of emission colors, long lifetime luminescence, large anti-Stokes spectral shifts, weak background autofluorescence, narrow emission bands, nonphotobleaching nature, blinking-free continuous emission capability, and relatively low toxicity [1,2,3]. So far, UCNPs with tailored multicolor emissions have underpinned a vast array of applications in energy conversion photovoltaics [4], fingerprint detection and anticounterfeiting barcoding [5,6], biosensing [7], super resolution nanoscopy [8], photodetectors [9], drug and gene delivery [10], photodynamic therapy [11], light-triggered on–off tattoo systems [12], photochemical tissue bonding [13] and so on. 

In particular, UCNPs have garnered increased interest in the quantitative detection of various biologically relevant targets, such as biomolecules, pH, ions, viruses, bacteria, reactive oxygen species and temperature [14]. However, the development of upconversion biosensing has been seriously hampered by poor NIR harvesting ability and a long-standing issue of quenching, which has multiple origin sources causing a great deal for scientists in finding the straightforward solution. At the edge of the UCNP surface, the excited state electrons undergo parasitic interaction with the surface defects (e.g., stacking faults, vacancies, dislocation, dangling bonds) which deplete the excitation energy leading to a partial decrease or complete “turn off” of the UCNP luminescence. 

Composition tailoring strategies by architecting the core with single or multiple shell structures are at the forefront to combat quenching issue. However, luminescence producing dopants are still kept at low doping levels to support quenching stability at the expense of upconversion brightness. In fact, quenching issue and composition limits can be addressed by extremely high NIR laser excitation powers (~10^6^ W/cm^2^ [15,16]), of which the main concern is associated with harmfulness and inapplicability for biomedical applications [17]. NIR laser power densities should be compatible with the tolerances of the skin tissue and kept below the maximum permissible exposure (MPE) values calculated according to ANSI Z136.1-2007 American National Standard for the Safe Use of Lasers [18]. The calculated MPE values for the corresponding wavelength of the NIR lasers are presented in Table 1.

The Förster (or fluorescence) resonance energy transfer (FRET) process has vast implications in quantitative and sensitive biosensing applications. In principle, an efficient FRET scenario occurs under specified conditions. First, there should a large degree of overlap in the emission-excitation profiles of the donor (excited state) and acceptor (ground state). Second, the long-range nonradiative dipole-dipole interaction between the donor-acceptor pair should be within the effective range of 10 nm [20]. The FRET process can be utilized for tuning optical properties of UCNPs by sizeable enhancement or depletion of their excitation or emission energy. Therefore, the FRET-based upconversion mechanism has been extensively exploited for bioanalytical sensors and assays, which enlivened modern bioimaging and biosensor research [21,22,23]. To date, extensive efforts have been focused on developing various energy acceptors, such as plasmonic nanostructures and organic dyes, which can aid in modulation photoluminescence efficiency of energy donating UCNPs via the FRET process. Compared to organic dyes which are prone to photodegradation, plasmon-enhanced upconversion has been considered as a breakthrough engineering strategy to minimize quenching issue, maximize upconversion efficiency and stimulate NIR harvesting offering unprecedented biosensing precision. In particular, the plasmon resonances could serve to achieve control over the luminescence properties of UCNPs by intentional amplification or quenching of the specific emission band [24], which could be beneficial for designing sensing platforms with specificity, low detection limits and linear response to the presence of the target bioanalyte. A recent literature survey has shown that several reviews related to the biosensing applications of UCNPs were published recently [7,14,25,26]. On the other hand, a focused overview of plasmon-modulated upconversion biosensing is still not available in the literature. Therefore, the objective of this review is to offer the reader a brief overview of the up-to-date strategies to incorporate plasmonic nanoparticles to lanthanide UCNPs for potential biosensing applications. 

## 2. Characteristics of UCNPs

### 2.1. Lanthanide Upconversion Basics: Mechanism and Origin

The upconversion is a nonlinear optical effect which gained widespread scientific interest with the advent of lasers. It is generally acknowledged that the concept of upconversion was proposed in the 1950s by Bloembergen and further developed by Auzel in the 1960s who introduced groundbreaking theoretical studies on the energy transfer upconversion process [27]. Since the 1990s, the emergence of nanoscience has brought a huge diversification of nanofabrication technologies leading to efficient downscaling upconversion phosphors to nanometer regime making them tremendously attractive across a range of biomedical applications [28]. 

Even now, the field of upconversion keeps evolving. Over time, it is believed to practically benefit health care and different industrial sectors. Ongoing research in the upconversion field continues to uncover and explain the complicated mechanisms behind photon upconversion. Meanwhile, there are several known mechanisms responsible for upconversion origins, such as excited state absorption, energy transfer upconversion, cooperative upconversion, photon avalanche, and energy migration upconversion [2]. This review deals with energy transfer based upconversion, therefore another deep-in depth description of upconversion mechanisms is beyond the scope of this review.

Upconversion in lanthanides originates from their ladder-like arrangement of energy levels which impart effective absorption and subsequent retaining of incident photons from external laser until a sufficient quantity is reached to proceed with upconverted emission. In principle, the upconversion process relies on electronic transitions within partially filled 4f orbitals. Notably, 4f shells in lanthanides are well protected by 5s and 5p shells (secondary electrons) rendering significant advantages, such as high resistance to (environmental influences) photobleaching, photochemical stability and sharp emission bands from 4f-4f transitions. However, the electron shield impedes 4f transitions and imposes limitations on light harvesting properties leading to poor absorption cross sections of lanthanides [29]. For a single and isolated lanthanide ion, these transitions are forbidden by selection rules of quantum mechanics, but this situation drastically changes when lanthanides are embedded as dopants inside the host matrix enhancing the probability of 4f-4f transitions. Selection rules on the spin are relaxed by a larger spin-orbit coupling. In particular, doping distorts site symmetry, which forms a stronger crystal field around lanthanide ions. Such absence of inversion symmetry (noncentrosymmetric system) distorts the electron cloud leading to intermixing with f states. As a result, Coulombic interactions and spin-orbit coupling of 4f subshell electrons lead to abundant energy sublevels and a large number of emitting levels [30]. 

### 2.2. Lanthanide Upconversion Composition

The most common configuration of lanthanide based UCNPs is composed of lanthanide ion dopants, such as sensitizers, emissive activators, and sometimes energy migration ions, that reside in the crystal host lattice. The concentration and distance between the sensitizer and emitter ions are critical parameters for tailoring the optical properties of UCNPs. In particular, higher concentration and greater proximity are essential to increase luminosity, but also invoke non-radiative cross-relaxations, known as the "concentration quenching effect". Presently, as the main strategy for overcoming the concentration quenching hurdle, the doping levels of sensitizer (~20%) and emitter ions (<2%) are kept at low percentages to optimize the luminescence brightness [31].

Host and dopants interaction occur in the form of energy exchange. Thus, some energy is absorbed by the host through vibrational coupling, which explains the choice of host lattices with the lowest phonon energy. Decades of research in the upconversion field have spawned an extensive array of host nanomaterials, such as various metal oxides, oxysulfides, vanadates, halides, phosphates and so on [32]. Among them, the most popular host is hexagonal phase β-NaYF_4_, which meets the requirements for ideal host materials, such as the lowest phonon frequency, high optical transparency, less hygroscopic and brighter emission than lanthanide fluoride (LnF_3_) host [33,34,35]. Sodium ion (Na^+^) has been widely introduced into the lanthanide fluoride lattice because it has nearly the same ionic radius as lanthanide ions [36]. For instance, Ayadi et al. reported that introducing Na^+^ ions into GdF_3_ led to lattice expansion and imparted stabilization of its hexagonal crystal structure [37]. For simplicity, this review specifically concentrates on plasmon modulated upconversion systems constructed using metal lanthanide fluoride hosts. 

**Figure 1:**
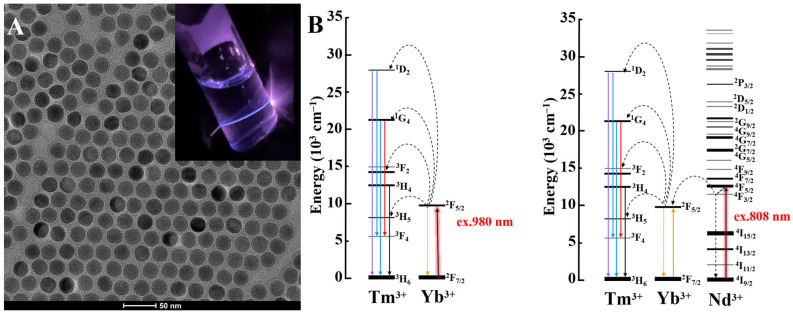
(**A**) Representative TEM image of NaYF_4_:Yb,Tm UCNPs (scale 50 nm) with digital inset of their UC emission under NIR laser. (**B**) Upconversion process of Tm^3+^ activator under 980 nm and 808 nm excitation.

Activator ions, as their name suggests, receive the excitation energy from the sensitizer or energy migration ion to activate emission. The choice of activator ion plays a decisive role in finely tuning emission profiles of UCNPs, for instance, erbium (Er^3+^) ion is recruited to produce red/green emissions, while thulium (Tm^3+^) is broadly exploited for obtaining UV/blue emissions from UCNPs (Figure 1A) [5]. Figure 1A exemplifies a typical transmission electron micrograph of NaYF_4_:Yb,Tm UCNPs. Digital inset illustrates visible to naked-eye blue emission of NaYF_4_:Yb,Tm UCNPs under NIR laser illumination. Popular activators, such as erbium (Er^3+^), thulium (Tm^3+^) and holmium (Ho^3+^) ions, possess narrow energy gap of 2000 cm^−1^. Thereby, they are more efficient for the energy transfer process compared to other luminescent ions with a wide energy gap of 7000 cm^−1^, e.g., terbium (Tb^3+^), europium (Eu^3+^) and dysprosium (Dy^3+^) which are prone to detrimental nonradiative energy losses. Activators, in general, have a complicated electronic structure and extremely small absorption cross-sections of ~10^−21^ cm^2^ responsible for a low quantum yield (<1%) and necessary in photoactivation using high laser power densities [38]. 

Sensitizers serve to accumulate incoming NIR photons and further transfer these photons to the activator ions directly or indirectly through energy migration ions. For example, ytterbium (Yb) is utilized as a ~980 nm NIR sensitizer with a single transition ^2^F_7/2_–^2^F_5/2_ levels, which showcases efficient energy transfer when paired with Tm^3+^ activator. Moreover, co-doping Yb ion with neodymium ion (Nd^3+^, <1 mol.%) allows tailoring 808 nm NIR harvesting upconversion system properties in UCNPs, where Yb^3+^ act as a migration ion facilitating the energy extraction from Nd^3+^ ions and its further transfer to activator ions [39]. Figure 1B displays the schematic energy diagrams of Tm^3+^ ion upconverted emission photoactivation under 980 nm and 808 nm NIR stimulation. Table 2 shows common lanthanide sensitizers and their corresponding absorption cross sections. 

Importantly, 808 nm NIR harvesting UCNPs have arisen as a feasible platform for biomedical application to overcome the safety shortcomings of those excitable under conventional 980 nm lasers, since water molecules absorption of 808 nm photons is 20 times weaker than 980 nm ones [31]. 

Over the past few decades, explosive growth in the development of nanotechnology offered numerous synthetic protocols and optimized designs of UCNPs. By now, several routes to synthesize UCNPs have been proposed and widely utilized, such as co-precipitation [28], thermal decomposition [43], hydrothermal method [44], microwave-assisted synthesis [45], microemulsion method [46] and the liquid–solid solution (LSS) process [47]. Among these synthesis methods, co-precipitation and thermal decomposition methods have found remarkably broad utility in the scientific community for the fabrication of highly monodisperse and bright upconversion nanocrystals. However, excitement over developments in the controlled synthesis of small UCNPs is moderated by luminescence intensity decrease as the size shrinks the quenching issue becomes more prominent. Especially, the enhanced surface-to-volume ratio leads to greater exposure of the UCNPs surface to external quenchers, such as OH impurities, and organic ligands C-H and C-C bonds with a high vibrational energy that present in the dispersion solvent or capping ligands [48,49]. The main strategy so far to remove surface quenching relies on adopting surface passivation with single or multiple outermost shell layers, which mitigate surface defects and preclude the interaction with the external quenchers. However, the core-shell structure fabrication process usually requires prolonged and multi-step synthetic procedures. Therefore, researchers in the upconversion field are faced with the need to accelerate (e.g., by continuous process [50]) or even automate the synthesis process [51]. 

## 3. Principles and Applications of Photon Modulation in Upconversion-Based Biosensors

**Figure 2:**
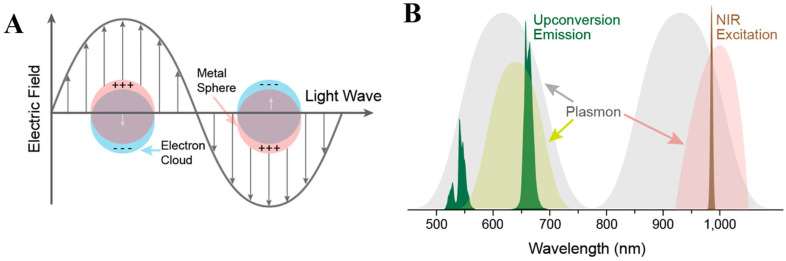
(**A**) Schematics of a metal nanoparticle’s electron cloud oscillation. (**B**) Spectral overlap between metal nanoparticle’s SPR and upconversion nanoparticles emission or excitation profile. Reprinted with permission from reference [53].

Surface plasmon resonance (SPR) is a light-matter interaction phenomenon inherent to materials with a negative real and small positive imaginary dielectric constant. When incident light interacts with the electron cloud of these materials, electrons start collectively oscillating inducing resonant effect (Figure 2A). Accordingly, surface plasmons could be localized or propagating. In the case of the localized surface plasmons, the excitation wavelength is larger than the nanoparticle causing its electron oscillation in a localized manner. Usually, the evanescent field forms the propagating surface plasmon (also known as surface plasmon polariton), in particular, when the incident light is polarized leading to surface charge oscillation along the metal/dielectric interface in the longitudinal direction and subsequently decay [52].

### 3.1. A Brief Theoretical Principles of Plasmon Modulated Upconversion 

SPR extension in UCNPs using plasmonic nanoparticles (PNPs) can serve to augment the functionality of upconversion sensing platforms by resonating its excitation or emission (Figure 2B) [53]. For example, upon plasmon-light coupling energy confinement leads to the amplification of the electromagnetic field benefiting NIR light harvesting abilities and radiative decay rates of UCNPs. Theoretically, plasmonic modulation can afford an enhancement of the upconversion emission intensity E^2n^ time without energy transfer alteration. So far, 100-fold enhancements were verified experimentally [54]. 

Thus, PNPs are exploited as optical nanoantennae that could also provide modulation over the radiation features of UCNPs through excitation energy redistribution leading to luminescence enhancement (Purcell effect) or intentional quenching [24,55].

The design of plasmon-mode upconversion nanoprobes is commonly based on the matched SPR band of plasmonic nanoparticles and emission/excitation profiles of UCNPs. For example, the aspect ratio of gold nanorods governs the SPR band location and hence could be easily adjusted to modulate upconversion process [56]. Noteworthy, the proximity and isolation between UCNPs and PNPs are critically important for efficient energy transfer and sustaining undesired quenching [57]. For example, the use of a silica shell as a spacer between UCNPs and PNPs enables to prevent of their direct contact and unintentional quenching [58]. Meanwhile, the plasmon-upconversion interaction considerably depends on the geometrical morphology of PNPs. For instance, a variety of plasmonic nanostructures, such as nanoshells, nanofilms, nanowires, nanorods and nanoparticle arrays/cavity, were coupled to UCNPs to achieve plasmon modulated upconversion [59]. 

Gonzalez et al. investigated the upconversion quenching ability of different sizes of Au NPs ranging from 3.9 nm to 66 nm. Their findings suggest that size range Au NPs between 15 and 20 nm is optimal to initiate intentional quenching in silica coated UCNPs. Conversely, larger Au NPs with the size >50 nm tend to amplify the upconversion emission. In addition, their study also elucidated that an optimal shell thickness for upconversion enhancement is ca. 12 nm, while efficient quenching demands the thinnest possible shell [60]. 

By exploiting plasmon modulated upconversion strategy, a range of nanoprobes was constructed and proposed for examining a broad variety of analytes in biological environments. We analyzed recent literature published on the practical implications of plasmonic modulation of upconversion biosensors to detect diverse analytes and compiled our review in Table 3, which serves to provide insights into key parameters for designing plasmon modulated upconversion nanoprobes, such as (1) the size and corresponding absorption (or SPR) band of PNPs; (2) the physicochemical and optical characteristic of UCNPs, such as the size, surface coating (spacer), NIR activation laser wavelength, emission profiles; (3) target analytes and achieved the limit of detection (LOD). In the following sections, we will highlight trending topics through discussion of some representative nanoprobes that are expected to provide a brief overview of the current research status in the plasmon-modulated upconversion biosensing field. 

### 3.2. Plasmon Modulated Upconversion Sensing of Ions and Small Biomolecules

Anion and cation recognition plays a crucial role to evaluate the operation of vital biological processes and garner information about overall cell health. There is a wealth of literature reporting the ion sensing platforms that utilize plasmon modulated upconversion, while capable of simultaneous measuring changes in the concentration of different small biomolecules. Therefore, we decided to combine the discussion on ions and small biomolecules sensing in one section. The detection principle is based on the ability of PNPs to modulate the radiation properties of UCNPs by disabling them in closer proximity and retrieving “turn on” them upon segregation. For example, Chen et al. constructed an assay for chromium (Cr^3+^) ion detection, which was composed of electrostatically coupled lysine-capped UCNPs and dimercaptosuccinic acid-capped gold nanoparticles (Au NPs). The presence of Cr^3+^ ions induces a drift away of Au NPs from UCNPs surface recovering the emission profiles in a linear response [61].

**Figure 3:**
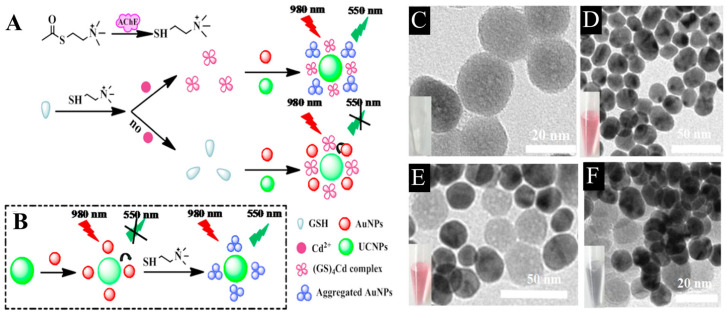
Sensing principle of the bifunctional UCNPs/AuNPs based nanoprobe for detection of (**A**) AchE and (**B**) Cd^2+^ ions with GSH regulation. Representative TEM images of (**C**) UCNPs, (**D**) AuNPs, (**E**) UCNPs/AuNPs, and (**F**) aggregation in UCNPs/AuNPs caused by post-addition of AChE and ATC. Reprinted with permission from reference [62].

Fang et al. developed a bifunctional biosensing platform using plasmon modulated upconversion effect of quantitative detection of acetylcholinesterase (AChE) and cadmium (Cd^2+^) ions with the help of glutathione (GSH). Two mechanisms account for the tendency of Au NPs to aggregate and drift away from UCNPs to recover the emission profile (Figure 3A,B). The first is that the presence of AChE promotes the hydrolysis of acetylthiocholine (ATC) which tend to destabilize the surface chemistry of Au NPs and lead to their aggregation (Figure 3C–F). The second mechanism relies on the ability of Cd^2+^ ions to detach GSH from the surface of Au NPs to form spherical shaped complex, which disrupts the stability of Au NPs and effectively triggers their gradual isolation from UCNPs. The designed biosensor exhibited LODs of 0.015 mU/mL and 0.2 µM for AchE and Cd^2+^ ions, respectively [62].

Hu et al. reported a bifunctional Au-UCNPs nanoprobe which exhibits a dose dependent response to Cd^2+^ ions and GSH with LODs 0.059 μM and 0.016 μM, respectively. In this study, the presence of GSH restrained the Au NPs aggregation, while co-existence Cd^2+^ ions impaired their stability leading to the gradual weakening of UCNPs red emission [63]. 

Sun and Gradzielski employed plasmon-modulated upconversion for the detection of poisonous cyanide ions. The sensor operation was based on the redox consumption of Au NPs by CN^−^ ions which enabled the emancipation of UCNPs to regain their luminescence and produce detection signal. Moreover, the nanoprobe demonstrated excellent selectivity and sensitivity by distinguishing cyanide ions from different interfering ions with a detection limit of 1.53 μM [66].

Remarkably, gold nanorods have gained great momentum from researchers around the world owing to their versatility in SPR band control via aspect ratio adjustment. For example, Kim et al. demonstrated 27-fold upconversion enhancement by altering the aspect ratio of Au NRs to match their SPR band with the emission of UCNPs at 805 nm. The UCNPs have been preliminarily encapsulated in polyamidoamine generation 1 (PAMAM G1) dendrimer, which served as a spacer to prevent undesired quenching upon coupling with Au NRs. The obtained nanoprobe was post-functionalized with 2-thiouracil for sensitive and selective detection of uric acid, which is an important small biomolecule. Because its elevated content may indicate renal or cardiovascular disorders in the human body [68]. Interestingly, Zhu et al. obtained 50-fold SPR based enhancement of the UCNPs brightness, which is associated with a ~24 nm thick silica layer on the surface Au NRs. Thereby proposing a nanoprobe for potential microRNA-21 detection in human serum samples and human breast cancer cell (MCF-7) lysates [67].

Noteworthy, the use of silver nanoparticles for the modulation upconversion biosensors has been relatively limited compared to gold nanoparticles, even though theoretically Ag NPs exhibit stronger SPR [85]. Among recent reports, Liu et al. employed ultrasmall (1.9 nm) silver nanoclusters as acceptors for the construction of plasmon modulated NIR upconversion nanoprobe for intracellular biothiols detection. The authors demonstrated the biosensor performance in the liver tissue of mice to highlight its potential for in vivo sensing [69]. 

### 3.3. Plasmon Modulated Upconversion Sensing of Biomacromolecules

**Figure 4:**
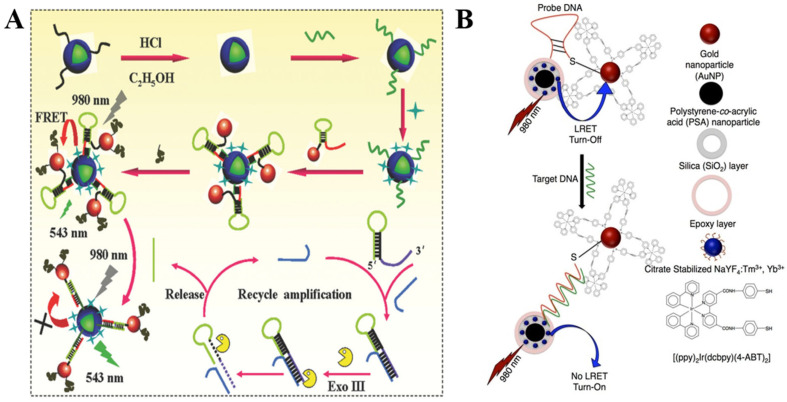
(**A**) Schematics of the UCNPs–AuNPs plasmon-modulated biosensing platform for highly sensitive detection of tumor-related ncRNA via the Exo III-assisted cycling amplification strategy. Reprinted with permission from reference [75]. (**B**) Schematics of the composition and energy transfer mechanism of ssDNA optical sensor composed of PSA/SiO_2_ coated UCNP and cyclometalated Ir(III)-AuNPs. Reprinted with permission from reference [73].

Over the past decade, our understanding of how to implement recognition of biomacromolecules, such as RNA or DNA nucleic acids, using plasmon modulated upconversion biosensing systems has significantly advanced. For example, recently, Zhu et al. reported a universal pathway for tumor related noncoding RNA (ncRNA) recognition (Figure 4A). The upconversion recovery principle in the designed biosensor is based on the uncoupling of DNA encapsulated UCNPs from Au NPs bearing a single molecule hairpin DNA (Hp) molecule via exonuclease III (Exo III)-assisted cycling amplification strategy. Notably, the biosensor exhibited a great sensitivity towards the expression level of miR-21 in human breast cancer cell (MCF-7) lysate with LOD of 0.54 fM [75].

Zourob et al. proposed an ssDNA target sequence sensing pathway containing blue-emitting UCNPs for incorporation into silica coated polystyrene-co-acrylic acid nanoparticles (PSA/SiO_2_) (donor) and Au NPs with immobilized Ir(III) complex (quencher), as shown in Figure 4B. In response to the sequential addition of the target DNA, the quencher could be effectively separated from the donor reactivating the upconversion signal under 975 nm NIR irradiation in a linear manner vs. DNA concentration. The developed sensor sensitivity was indicated at LOD as low as 1 pM. Furthermore, the selectivity of the DNA sensor was confirmed through the titration of the nanoprobe using the DNA conjugated nanohybrids with single base mismatch [73].

Conversely, some researchers employ the fluorescence quenching as a signal to recognize the analyte. For instance, Chen et al. employed a simple paper-supported aptasensor to construct Au NR/UCNPs based plasmon modulated nanoprobe for cancer biomarker exosome. The designed nanoprobes operation is activated in the presence of exosome. Upon conjugation with CD63 protein, Au NRs and UCNPs parts are brought to proximity enough to initiate linear quenching which is correlated with exosome concentration. The LOD of exosomes was estimated to be 1.1 × 10^3^ particles/μL [74].

### 3.4. Plasmon Modulated Upconversion Sensing of Viruses

Despite encouraging progress in the development of safe disease diagnostic tools, such as reverse transcription-polymerase chain reaction (RT-PCR) and enzyme-linked immunosorbent assay (ELISA), rapid and ultrasensitive bioassays for pathogenic viruses detection are still very demanding. So far UCNPs were utilized for the detection of H5N1 Influenza [86], oligonucleotide markers of the SARS-CoV-2 virus [87,88], hepatitis B Virus surface antigen (HBsAg) [89], anti-human immunodeficiency virus (HIV) antibodies [90], thrombocytopenia syndrome virus (SFTSV) total antibodies [91]. 

**Figure 5:**
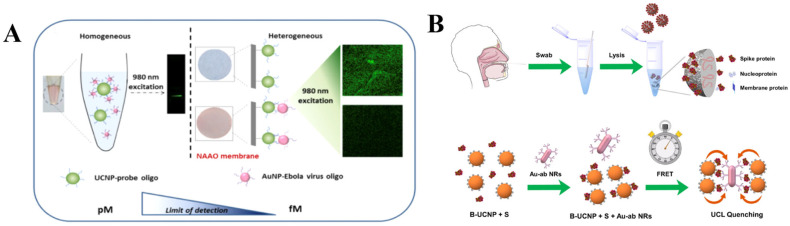
(**A**) Comparison illustration of homogenous and heterogeneous sensor for Ebola virus detection. Reprinted with permission from reference [80]. (**B**) Schematics of COVID S protein detection using plasmon modulated upconversion biosensing system. Reprinted with permission from reference [81].

There are several reports on deploying plasmon modulated upconversion for construction of novel rapid biosensors for virus recognition. For instance, in 2016, Hao et al. employed plasmon modulated upconversion for ultrasensitive detection of Ebola virus, which outbreak threatened the world significantly between 2014 and 2016 (Figure 5A,B). In their research, they proposed a bioassay composed of BaGdF_5_:Yb,Er UCNPs and Au NPs that were immobilized on the 3D structured nanoporous alumina (NAAO) substrate. The spectral overlap between nanoparticles allowed to observe effective quenching of naked-eye observable green UC emission upon the combination of probe oligoconjugated with UCNPs and Ebola virus oligoconjugated with AuNPs. Moreover, the authors demonstrated that their sensor could effectively detect Ebola viral RNA in clinical samples with LOD of 500 fM, which is comparable with other conventional detection methods [80]. 

In another investigation by Hao et al., they introduced plasmon modulated biosensing of SARS-CoV-2 spike protein for COVID-19 point-of-care diagnostics (Figure 5C,D). In this study, the NaYF_4_:Yb/Tm UCNPs nanoprobe was complemented with Au NRs, which effectively captured and detected S protein endowing LOD of 1.06 fg mL^−1^ [81].

Hepatitis B (commonly abbreviated as HBV) is a DNA virus that causes a serious damage to the human liver. It has been estimated that more than 300 million people around the globe are infected with HBV [92]. Researchers have devoted substantial efforts to the development of sensitive biosensing tools for the prevention of HBV infection spread. For example, Zhu et al. proposed to employ Au-UCNPs-based nanoprobes for HBV DNA detection. In this study, Au NPs were bound to UCNPs via DNA hybridization keeping upconversion emission quenched. The situation drastically changed upon introducing target DNA, which initiated the departure of Au NPs from the UCNPs surface with subsequent restoration of the emission intensity. The proposed nanoprobe exhibited a LOD of 250 pM [79]. 

### 3.5. Plasmon Modulated Upconversion Sensing of Temperature

**Figure 6:**
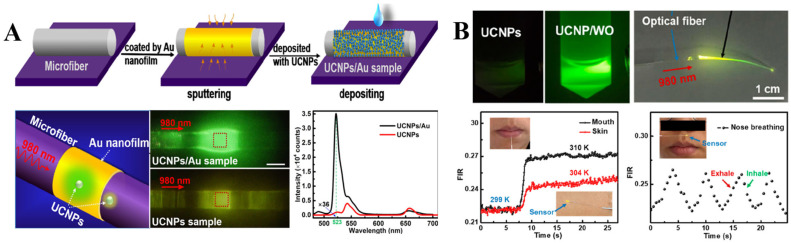
(**A**) Illustration of the selective plasmon-enhanced green emission upconversion nanoprobe for temperature sensing, which include: schematics of the fabrication process, optical images of the sensor upon laser excitation and comparative illustration of the upconversion photoluminescence enhancement induced by Au nanofilm. Reprinted with permission from reference [83]. (**B**) Optical images of UCNPs and UCNPs/WO solutions under a 980 nm laser illumination. Fluorescence intensity ratio changes from the air to the mouth (black line), hand skin (red line) and time-dependent breath sensor response (dotted line). Reprinted with permission from reference [84].

Temperature-dependent properties of UCNPs arising from complex thermally coupled energy levels are suitable for plasmon modulated thermometric biosensing. For example, Li et al. fabricated an optical microfiber (~3 μm) coated with an 18 nm thin gold film and decorated with UCNPs for temperature sensing (Figure 6A). The work principle of the constructed temperature sensor is based on the plasmon enhanced upconversion luminescence, which facilitated temperature dependent upconversion emission enhancement. In particular, plasmonic properties of Au nanofilm were activated by 980 nm laser which selectively increased the green emission intensity of UCNPs at 523 nm by 36 times, which is ascribed to the Au nanofilm-assisted local field enhancement of the incident light. Upon precise control of the UCNPs’ amount and laser dosage, the designed sensor can respond to the temperature range of 325–811 K with a resolution of 0.034–0.046 K [83]. 

Recently, Li et al. developed a flexible temperature sensor, consisting of polyacrylic acid (PLA) fiber and UCNPs/W_18_O_49_ upconversion/plasmonic semiconductor hybrid optical system for potential application in wearable health monitoring (Figure 6B). Co-doping W_18_O_49_ nanowires with UCNPs into PLA fiber with a high refractive index of 1.46 facilitated the plasmon enhanced upconversion green emission at 540 nm, which is suitable for ratiometric reading of the temperature changes under a 980 nm NIR laser excitation (5.5 mW). The hybrid sensor exhibited high sensitivity of 1.53% with 0.4 K LOD. In general, the ratiometric response could be measured based on the fluorescence intensity ratio of upconversion emission peaks produced by UCNPs under NIR laser excitation. In this study the intensity ratio between green emissions at 520 nm and 540 nm was used as a temperature change indicator, because these emissions originate from the thermally coupled energy levels ^2^H_11/2_ and ^4^S_3/2_ of Er^3+^ activator [84]. 

## 4. Future Directions

Continuing advances in the upconversion field opened the emerging frontiers of plasmon modulated biosensing technology, which affords background-free, rapid, well-timed and more sensitive quantitative data acquisition regarding bioanalytes compared to traditional sensing approaches. In this review, we covered the basic concepts of lanthanide upconversion and brief theoretical principles plasmon modulated upconversion, we systematically analyzed cutting edge advances in construction biosensing tools through the integration of plasmonic nanoparticles (predominantly gold nanoparticles) with upconversion nanoparticles for nanoprobing various biological analytes. 

Herein, we have reviewed ongoing research progress made on the development of plasmon modulated upconversion biosensors. Thus, from the most recent experimental findings we derived the future trends and perspectives to better understand how to improve UCNPs luminescence features using plasmonic nanoparticles. In this regard, to fully realize their potential in practical plasmon modulated upconversion biosensing, the following criteria still need to be fulfilled to meet exceedingly high standards of in vivo models and their further clinical translation: 

(1) The size of nanoparticles should be monodisperse and in the renal clearable range (<10 nm); however, the size should not be too small (<5 nm) to prevent too rapid excretion from the body. Another important consideration is that as the size of UCNPs decreases the brightness degrades dramatically because the quenching issue becomes more profound; 

(2) the stabilization of UCNPs in biological environments with minimized toxicity, since the dissolution in aqueous media has been a general drawback of NaYF_4_ host materials which leads to the release of cytotoxic lanthanide and fluorine ions. Moreover, plasmon modulation efficiency is sensitive to the nature and thickness of the spacer coating on UCNPs or PNPs. This aspect makes it more demanding to understand the nanoparticle functionalization process and seek alternative coatings which impart more efficient plasmon-enabled control of upconversion properties;

(3) high photoluminescence quantum yields are generally desirable for sufficient brightness under safe NIR excitation laser powers; 

(4) the use of 980 nm NIR laser based photoactivation remains a subject of debate, which is unlikely to be increasingly used in the future due to tissue overheating issues. We anticipate that the focus of the upconversion biosensing research will gradually shift toward safer and shorter NIR laser wavelengths, such as an 808 nm laser-stimulated biosensing, yet it has been restricted by a low permissible power dosage; 

(5) from the perspective of temperature-sensitive luminescence of UCNPs, there is little doubt about the rationality of their complementary use, since PNPs tend to generate heat under NIR laser irradiation. It is widely acknowledged that elevated temperatures accelerate the decay rates of thermally coupled energy levels in UCNPs leading to their poor upconversion luminescence or even undesired quenching. Though from our literature survey, it is clear that plasmon enhancement does improve the upconversion efficiency and benefit its biosensing performance. 

## 5. Conclusions 

The progress in plasmon-modulated upconversion biosensing will continue to advance, which may enable to explore alternatives to gold plasmonic nanostructure that can provide better plasmonic resonances. Yet, a more profound understanding of the exact key parameters (size, geometry, thickness of the spacer, etc.) governing the efficient plasmon enhancement is therefore essential. Overall, plasmon-modulated upconversion is envisioned as exciting and very promising avenue for the future development of intelligent biosensors that could possibly benefit the healthcare system. We hope this literature survey will motivate the reader to explore solutions to tackle the remaining problems and possibly confront the conceptual challenges to overcome the current bottleneck.

## Figures and Tables

**Table 1:** Calculated safe power densities of common NIR lasers [19] (MPE—maximum permissible exposure, NIR—near-infrared).

NIR laser, λ	808 nm	915 nm	980 nm	1064 nm
MPE value	~0.329 W/cm^2^	~0.538 W/cm^2^	~0.726 W/cm^2^	~1.0 W/cm^2^

**Table 2:** Common lanthanide sensitizers and their absorption cross-sections [40,41,42].

Sensitizer	Absorption Wavelength, λ	Absorption Cross-Section, cm^2^
Ytterbium (Yb^3+^)	980 nm	~10^−20^
Neodymium (Nd^3+^)	740 nm 800 nm 860 nm	~10^−19^
Erbium (Er^3+^)	1500 nm	1.1 × 10^−20^ cm^2^

**Table 3:** Photon-modulated upconversion nanoparticles (PNPs—plasmonic nanoparticles, —upconversion nanoparticles, λ abs.—emission wavelength, NIR—near-infrared, λ em.—emission wavelength, LOD—limit of detection).

PNPs	Size	λ abs.	UCNPs	Coating	Size	NIR Laser	λ em.	Analyte	LOD	Ref.
Au NPs	13 nm	522 nm	NaYF_4_:Yb20%,Er2%	lysine	70 nm	980 nm	540 nm	Cr^3+^	0.8 nM	[61]
Au NRs Au NBs	9–10 nm 15 nm	515/692 nm 525 nm	NaYF_4_:Yb20%,Ho2%, Mn	PAA	20–30 nm	980 nm	542 nm, 660 nm	Pb^2+^, Hg^2+^	50 pM 150 pM	[64]
Au NPs	20 nm	528 nm	NaYF_4_:Yb20%,Er5%	CTAB	20 nm	980 nm	-	Cd^2+^ Ache	0.2 μM 0.015 U/mL	[62]
Au NPs	15 nm	521 nm	NaYF_4_:Yb20%,Er2% @ NaYF_4_	SiO_2_ (2 nm)	37 nm × 28 nm	980 nm	522/545/654 nm	Cd^2+^ GSH	0.059 μM 0.016 μM	[63]
Ag NPs	12 nm	398 nm	NaY/GdF_4_:30%Yb20%,Er2%	NH_2_	32 nm	980 nm	545/660 nm	Cr^3+^	34 nM	[65]
Au NPs	1.7 nm	535 nm	NaYF_4_:Yb20%,Er2%@ NaYF_4_:Yb20%	PEI (8 nm)	43 nm	980 nm	655 nm	CN^−^	1.53 μM	[66]
Au NRs	80 nm × 25 nm	-	NaYF_4_@NaYF_4_:Yb20%,Er2% @ NaYF_4_	H1	10 nm	980 nm	543 nm	microRNA	0.036 fM	[67]
Au NRs	~45 nm	520 nm	NaYF_4_:Yb20%,Tm2%	PAMAM (~2.5 nm)	~90 nm	980 nm	450/470/805 nm	uric acid	1 pM	[68]
Ag NCs	1.9 nm	500/620 nm	NaYF_4_:Yb20%,Tm2%	PEI	30 nm	980 nm	480 nm	biothiols	-	[69]
Au NRs	-	-	NaYF_4_:Yb20%,Tm2%	PEI	27.7 nm	980 nm	656 nm	DNA methylation	7 pM	[56]
Au arrays	-	-	NaYF_4_:Yb25%,Tm0.3%	PAA	26 nm	980 nm	345/450/475/ 800 nm	Vitamin B12	3.0 nM	[70]
Au NPs	13 nm	521 nm	NaYF_4_:Yb20%,Ho2%	SiO_2_ (12 nm)	115 nm	980 nm	483/543/640 nm	ABA aptamer	3.2 nM	[71]
Au NPs	~50 nm	-	NaYF_4_:Yb20%,Er2%	PAA	~43 nm	980 nm	-	Aflatoxin B1	0.17 ng/mL	[72]
Au NPs	-	544 nm	NaYF_4_:Yb27%,Tm0.5%	SiO_2_/PSA	20 nm	975 nm	-	ssDNA	1 pM	[73]
Au NRs	27 nm × 54 nm	630 nm	NaYF_4_:Yb,Er	PEI	25 nm	980 nm	545/660 nm	Exosome	1.1 × 10^3^ part./μL	[74]
Au NPs	5 nm	~543 nm	NaYF_4_:Yb20%,Er2%@NaYF_4_	LDNA	21 nm	980 nm	543 nm	miR-21	0.54 fM	[75]
Au NPs	~50 nm	~530 nm	NaYF_4_:Yb20%,Er2%	PSA	~42 nm	980 nm	~550 nm	antibodies (Ab1)	2.3 pM	[76]
Au NPs	30 nm	520 nm	NaYF_4_:Yb20%,Er2%	Con-A	40–55 nm	980 nm	545/675 nm	glucose	0.02 μM	[77]
Ag NPs	7.8 nm	434 nm	NaYF_4_:Yb30%,Tm0.5%@NaYF_4_	bared	24 nm	980 nm	345/360/450/ 474 nm	glucose H_2_O_2_	1.41 μM 1.08 μM	[78]
Au NPs	11.9 nm	540 nm	NaYF_4_:Yb18%,Er2%	PEI	48 nm	980 nm	543/656 nm	Hepatitis B HBV DNA	250 pM	[79]
Au NPs	-	523 nm	BaGdF_5_:Yb20%,Er2%	NAAO	14 nm	980 nm	523/546/654 nm	Ebola	500 fM	[80]
Au NRs	~78 nm × 15.5 nm	965 nm	NaYF_4_:Yb11.9%,Tm0.1%	PEI	~30 nm	980 nm	480/800 nm	COVID S protein	1.06 fg/mL	[81]
Au NPs	20 nm	400–700 nm	NaYF_4_:Yb,20%Er2%	Apt2	35 nm		525/545/650 nm	Shigella	30 CFU/mL	[82]
Au nanofilm	18 nm	980 nm	NaYF_4_:Yb20%,Er2%	microfiber	38 nm	980 nm	523/545/655 nm	T(K)	325 K–811K	[83]
W_18_O_49_	100–800 nm × 5–30 nm	600–1400 nm	NaYF_4_:Yb2%@NaYF_4_:Er20%	PLA fiber	35 nm	980 nm	520/540/654 nm	T(K)	298 K–358 K	[84]

## Data Availability

Not applicable.

## Abbreviations

AChE	acetylcholinesterase
Con-A	concanavalin A
CTAB	cetyl trimethyl ammonium bromide
COVID	coronavirus disease
DNA	deoxyribonucleic acid
FRET	fluoresecence resonance energy transfer
GSH	gluthathione
HBV	Hepatitis B virus
Ln	Lanthanide
LOD	limit of detection
NAAO	nanoporous alumina
NCs	nanoclusters
NDs	nanodots
NPs	nanoparticle
NIR	near-infrared
NSs	nanosheets
PAA	polyacrylic acid
PAMAM	polyamidoamine
PEI	polyethylene imine
PLA	polyacrylic acid
PNPs	plasmonic nanoparticles
PSA	polystyrene-co-acrylic acid
RNA	ribonucleic acid
SPR	surface plasmon resonance
TEM	transmission electron microscopy
UCNPs	upconversion nanoparticles
